# Formation of Multicolor Nanogels Based on Cationic Polyfluorenes and Poly(methyl vinyl ether-alt-maleic monoethyl ester): Potential Use as pH-Responsive Fluorescent Drug Carriers

**DOI:** 10.3390/ijms22179607

**Published:** 2021-09-04

**Authors:** Marta Rubio-Camacho, María José Martínez-Tomé, Amalia Mira, Ricardo Mallavia, Carmen Reyes Mateo

**Affiliations:** Instituto de Investigación, Desarrollo e Innovación en Biotecnología Sanitaria de Elche (IDiBE), Universidad Miguel Hernández, 03202 Elche, Spain; marta.rubioc@umh.es (M.R.-C.); a.mira@umh.es (A.M.); r.mallavia@umh.es (R.M.)

**Keywords:** nanogels, PMVEMA, fluorescent conjugated polymers, doxorubicin, nanoparticles, drug-delivery, bioimaging

## Abstract

In this study, we employed the copolymer poly(methyl vinyl ether-alt-maleic monoethyl ester) (PMVEMA-Es) and three fluorene-based cationic conjugated polyelectrolytes to develop fluorescent nanoparticles with emission in the blue, green and red spectral regions. The size, Zeta Potential, polydispersity, morphology, time-stability and fluorescent properties of these nanoparticles were characterized, as well as the nature of the interaction between both PMVEMA-Es and fluorescent polyelectrolytes. Because PMVEMA-Es contains a carboxylic acid group in its structure, the effects of pH and ionic strength on the nanoparticles were also evaluated, finding that the size is responsive to pH and ionic strength, largely swelling at physiological pH and returning to their initial size at acidic pHs. Thus, the developed fluorescent nanoparticles can be categorized as pH-sensitive fluorescent nanogels, since they possess the properties of both pH-responsive hydrogels and nanoparticulate systems. Doxorubicin (DOX) was used as a model drug to show the capacity of the blue-emitting nanogels to hold drugs in acidic media and release them at physiological pH, from changes in the fluorescence properties of both nanoparticles and DOX. In addition, preliminary studies by super-resolution confocal microscopy were performed, regarding their potential use as image probes.

## 1. Introduction

The development of biocompatible nanosystems that integrate increasingly sophisticated functions, such as imaging and therapeutic properties, in one entity is having a high impact in pharmaceutical and biomedical applications, establishing smart nanomedical vehicles with theragnostic applications [1,2,3,4,5]. The main component of these multifunctional nanoplatforms is the carrier, which is responsible for the transport and delivery of the drug. Polymers are extensively used to this end, due to their great versatility and biocompatibility, and their ability to encapsulate, adsorb and covalently bind drugs and penetrate through biological barriers and reach the target site effectively [6,7]. In addition, these polymeric nanocarriers can be further modified as stimuli-responsive systems based on triggered release mechanisms (pH, ionic strength, electric field, temperature, light, etc.) to achieve maximal therapeutic efficacy [8]. Nanogels, which possess characteristic properties like swelling, responsiveness and softness as well as high drug encapsulation capacity, tunable size, ease of preparation, minimal toxicity and stability, are an example of it [9,10].

**Scheme 1:**
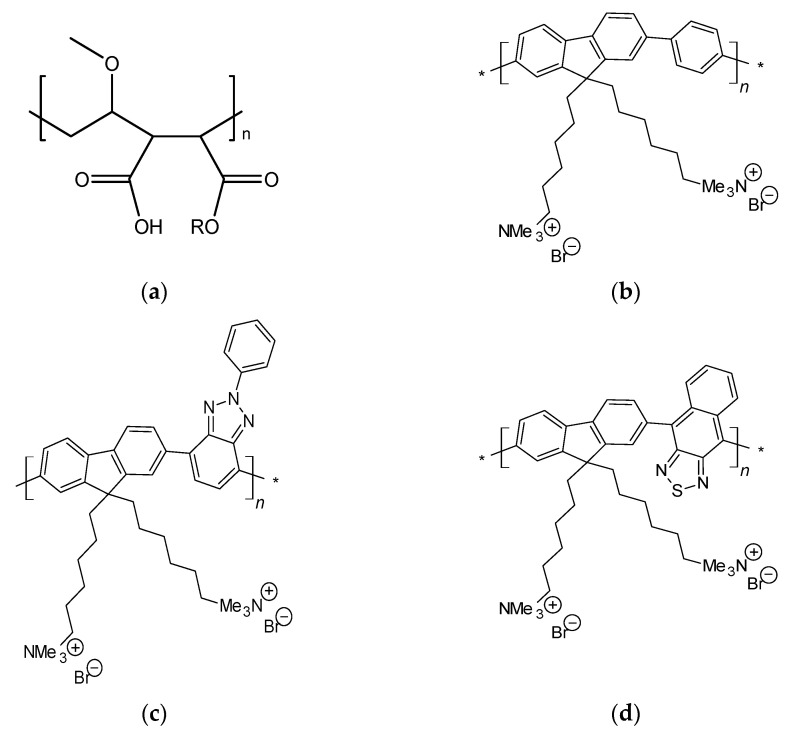
Chemical structures of the polymers (**a**) PMVEMA-Es and the CPEs (**b**) HTMA-PFP, (**c**) HTMA-PFBT and (**d**) HTMA-PFNT.

Polyanhydrides represent an important class of polymers that has been used for medical purposes including encapsulation of drugs in nanoparticles [11,12,13]. They can be prepared easily from available, low-cost resources and can be manipulated to meet desirable characteristics [14,15]. Furthermore, polyanhydrides have inherent high reactivity toward water and degrade into dicarboxylic acids, which are eliminated from the body as metabolites, rendering them highly biocompatible [16]. Examples of these biodegradable materials include poly(methyl vinyl ether-alt-maleic anhydride) (PMVEMA), an alternating linear copolymer of the monomers methyl vinyl ether and maleic anhydride which presents suitable properties as high biocompatibility and mucoadhesivity [17,18]. Its usefulness as a drug carrier in the treatment of various diseases has been described in recent years, especially for oral drug administration [19]. Moreover, it has been used as starting material for making biocompatible hydrogels with very good swelling capability, excellent mechanical and adhesive properties [20,21]. The anhydride group of PMVEMA can easily react with a large number of nucleophilic groups such as water, alcohols, acids and bases, which allows it to form different derivatives with different properties, among which it is worth mentioning the poly(methyl vinyl ether-alt-maleic monoethyl ester) (PMVEMA-Es) (Scheme 1). This derivative stands out for its biodegradability, bioavailability, biomucoadhesivity, low toxicity and price and is used in cosmetic and commercial oral hygiene products, mainly as a film former and adjuvant in transdermal patches [22]. Recently, our group has used PMVEMA-Es to fabricate drug-loadable electrospun nanofibers [12,23], but its use as a carrier in the preparation of multifunctional nanoplatforms has scarcely been considered so far [13].

The incorporation of bioimaging agents into the polymeric nanoparticles increase their functionality, providing useful information for the diagnosis of disease while monitoring the pathway of the drug, and its final location [8,24,25]. Among the various imaging methods that have been developed, fluorescence imaging is widely used because of its advantages of technical simplicity, high sensitivity, selectivity to a particular guest component/molecule and non-invasiveness [26]. In addition, if two or more fluorophores possessing different emission wavelengths are used, multicolor image can be obtained [27]. Organic dyes, fluorescent proteins and Quantum Dots (QDs) are the most commonly used markers to provide nanoparticles with fluorescent properties. These compounds exhibit interesting properties, but also important disadvantages. For example, organic dyes and fluorescent proteins often suffer from photobleaching and poor photochemical stability whereas QDs generally include heavy metals (cadmium and selenide) which are toxic to living organisms [26,28]. Compared to these fluorescent markers, conjugated polyelectrolytes (CPEs) provide a unique set of properties, including high fluorescent quantum yields, good photostability, broad absorption spectra, tuneable band gap, good photostability and biocompatibility and ease of bioconjugation [29]. These polymers are versatile functional materials characterized by a backbone chain of alternating double and single bonds containing pendant ionic groups which facilitate their water solubilization and their coupling with ions and biological systems, via electrostatic and hydrophobic interactions [30,31]. The versatility in their synthesis allows a fine-tuning of their absorption and emission spectra through backbone modification as well as the control over solution processability by changes in the ionic groups. Given these interesting properties, CPEs are considered fluorescent probes for bioimaging with enormous potential in diagnostic and therapy [32,33,34].

Our group has synthesized and characterized three fluorene-based cationic CPEs, that can emit fluorescence with high quantum yield and photostability in the blue, green and red regions of the visible spectrum: copoly-((9,9-bis(6′-N,N,N-trimethylammonium)hexyl)-2,7-(fluorene)-alt-1,4-(phenylene)) bromide (HTMA-PFP), copoly-((9,9-bis(6′-N,N,N-trimethylammonium)hexyl)-2,7-(fluorene)-alt-4,7-(2-(phenyl) benzo(d) (1,2,3) triazole)) bromide (HTMA-PFBT), and copoly-((9,9-bis(6′-N,N,N-trimethylammonium)hexyl)-2,7-(fluorene)-alt-1,4-(naphtho(2,3c)-1,2,5-thiadiazole)) bromide (HTMA-PFNT) (Scheme 1). These polyfluorenes show interesting properties as fluorescent markers for bioimaging and sensing devices and have recently been used to develop multicolor fluorescent liposomal nanoparticles activated by temperature, capable of being excited at the same wavelength [35,36,37,38,39,40,41]. In the present work, we take advantage of the attractive properties of CPEs, together with those of PMVEMA-Es and our previous experience in both types of polymers, to prepare and characterize fluorescent nanoplatforms with emission in different visible bands and potential applications as drug carriers and multiplexed bioimaging agents. The size, zeta potential, polydispersity, morphology and fluorescent properties of these nanoparticles have been characterized, as well as their stability under different conditions. Because the of PMVEMA-Es contains an ionizable acidic group in its structure (a carboxylic acid group) with a pKa ~5.3, we also explored the effect of pH on the new formulations, finding that the nanoparticles have the properties of a reversibly cross-linked system, as their overall size is responsive to pH and ionic strength, swelling at physiological pH and returning to their initial size at acidic pHs. Therefore, this type of polymeric nanostructures can be regarded as anionic nanogels [42,43]. Previous studies have reported the stability of anionic nanogels at pH lower than the physiological one and their application to protect sensitive drugs from the acidic conditions of the stomach. For instance, they have been used for more efficient release of dexamethasone in the colon and to improve insulin oral bioavailability [44,45,46]. In the present work, we explored the ability of the developed fluorescent nanogels to hold drugs in acidic media and release them at physiological pH, using doxorubicin (DOX) as a model drug. In addition, preliminary studies by super-resolution confocal microscopy were performed, concerning their potential use as image probes, using giant unilamellar vesicles as cell membrane models.

## 2. Results and Discussion

### 2.1. Characterization of PMVEMA-Es NPs

Nanoparticles composed of PMVEMA-Es (0.03 M in terms of repeat units) were prepared in water, following the protocol described in Materials and Methods, and subsequently characterized. Table 1 shows the result of the measurement of the hydrodynamic diameter (d), polydispersity index (PDI) and Zeta Potential (ZP) of the obtained NPs. The low PDI value, around 0.1, indicates that NPs are monodisperse, while the negative ZP value, close to −30 mV, suggests the existence of some deprotonated carboxyl groups on the NPs surface and guarantees adequate colloidal stability of the aqueous suspension. Such feature favours biocompatibility, since several studies have confirmed that nanoparticles with negative surface charge are safer and more suitable than the positively charged ones for in vivo applications [47,48].

The size and morphology of the polymeric NPs were also explored by TEM (Figure S1). Results indicate that nanoparticles are rather spherical with a size in agreement with those obtained from DLS experiments. Furthermore, long-term stability was explored by measuring the evolution of the hydrodynamic diameter over 30 days at 4 °C. Results are shown in Figure S2 and reveal no significant changes in the size over time, which confirms ZP results. Similar results were obtained when glucose or sucrose (0.2 M) were added to the aqueous solution (Figure S2A), which is information of interest for fluorescence microscopy assays to be described later. The temperature effect was also explored by DLS, showing that changes in the studied range, between 10 and 50 °C, did not modify the size of NPs (Figure S2B).

**Figure 1:**
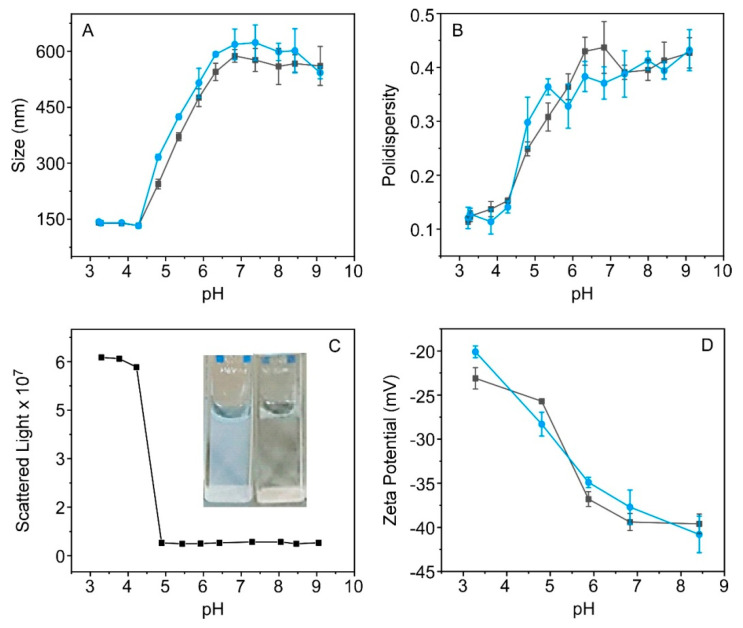
Effect of pH on (**A**) the hydrodynamic diameter, (**B**) polydispersity, (**C**) scattered light and (**D**) Zeta Potential of PMVEMA-Es NPs (0.03 M) in the absence (black points) and in presence of HTMA-PFP (1.5 µM) (blue points). Inset in Figure 1C shows a picture of the NP suspension at pH 4 (left) and pH 7 (right).

Contrary to what was observed with temperature, pH changes had a marked effect on the final size of NPs. As shown in Figure 1A,B, the hydrodynamic diameter and polydispersity of NPs showed an approximately sigmoidal response to pH. These parameters remained stable below pH 4.5, but increased significantly between pH 5 and 7, reaching their maximum values (~600 nm and PDI ~0.4) at pH values equal to or greater than 7. It was accompanied by a change in the visual appearance of the sample, from a turbid dispersion at acidic pH to a transparent solution at physiological pH (inset in Figure 1C). We also analyzed the light scattered by the NP suspension as a function of pH (Figure 1C). This experiment was directly made in the spectrofluorometer, by selecting the same wavelength for both excitation and emission monochromators with the smallest slits. The scattered light (430 nm) was collected at an angle of 90° of the incident light. As is shown in Figure 1C, a sharp drop of the scattered light was observed around pH = 5, which coincides with the increase in the volume of the nanoparticles. This behaviour is characteristic of hydrogel nanoparticles (or nanogels), which suffer large-magnitude volume phase transition in response to changes in environmental conditions and suggests that PMVEMA-Es NPs can be considered as such [49]. The reduction in the turbidity of the sample reveals the drastic decrease in the density of the nanoparticles after swelling, which could be a consequence of the deprotonation of the carboxylic group of PMVEMA-Es. Since the pK_a_ value of this group is ~5.3, at pHs above this value the polymer chains will become more negatively charged (not only on the nanoparticle surface) so they will repel each other, causing their stretching and leading to an increase in the volume of the nanoparticle (the water content increases) and a decrease in its density. In contrast, at pH < pK_a_, the PMVEMA-Es chains become more hydrophobic, which favors the creation of polymer chain–chain interactions that, being stronger than chain–solvent interactions, induce chain aggregation and cause shrinkage of the nanoparticle. The ZP values measured at different pHs support this hypothesis. This parameter became more negative when going from pH 3.2 (~−22 mV) to pH 7 (−40 mV), remaining stable at higher values (Figure 1D). The nanoparticles underwent a reversible swelling–shrinking behaviour as the pH decreased from 9 to acidic values, and the size and initial values of PDI and ZP were practically recovered (Figure S3).

It is known that the ionic strength of the solution also affects the swelling of nanogels. In the collapsed state, the effect of ionic strength is minimal, however, at a pH > pK_a_, increasing the ionic strength causes ion shielding that diminishes the degree of electrostatic repulsion of the negative carboxylic acid groups, reducing the size of the nanogel [50]. In order to explore this behavior, increasing concentrations of NaCl were added to a solution containing NPs of PMVEMA-Es at pH 7.4, and their size was measured by DLS. Figure S4 shows how the size decreased from 600 to 300 nm, as the ionic strength of the medium increased, which again evidences the nanogel-like behavior of PMVEMA-Es NPs.

### 2.2. Design and Characterization of Fluorescent PMVEMA-Es NPs

Once the PMVEMA-Es NPs were characterized, the next step was to obtain fluorescent polymeric NPs. To this end, we selected three fluorene-based cationic conjugated polyelectrolytes: HTMA-PFP, HTMA-PFBT and HTMA-PFNT, which incorporate in the fluorene backbone, respectively, a phenyl group, the chromophore 2-phenylbenzotriazole and the naphtho(2,3c) (1,2,5)thiadiazole group and emit in the blue, green and red region of the visible spectrum (Scheme 1). In previous studies of our group, we reported that these polyfluorenes show a very low fluorescence quantum yield in aqueous solutions, which is attributed to the formation of metastable aggregates by self-assembly of their hydrophobic chains and to the electrostatic interactions between the aggregates and the anionic species contained in the solution. In contrast, in presence of lipid vesicles, a strong increase in the fluorescence intensity and a blue-shift of the spectrum is observed, especially for HTMA-PFP, which evidences the breaking of aggregates as a consequence of the interaction and insertion of the polymer chains in the lipid bilayer [34,35,36,38]. With these results in mind, we explored the ability of these polyfluorenes to be incorporated into the PMVEMA-Es NPs by monitoring the changes in their fluorescence intensity. This study was performed fundamentally with the blue-emitting polyelectrolyte HTMA-PFP, in order to establish the optimal conditions for the preparation of fluorescent NPs and to characterize their properties.

#### 2.2.1. Interaction of HTMA-PFP with PMVEMA-Es and Formation of Blue-Emitting NPs

**Figure 2:**
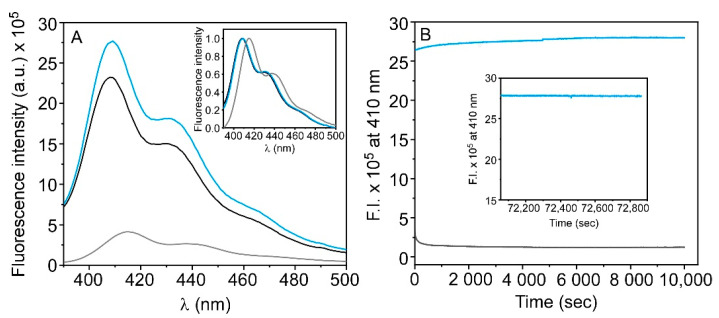
(**A**) Fluorescence emission spectra of HTMA-PFP (1.5 µM) in water (gray) and in PMVEMA-Es NPs (0.03 M), added during (black) and after (blue) the synthesis process. Inset: Normalized fluorescence emission spectra. (**B**) Time-dependence of the fluorescence intensity of HTMA-PFP, measured at 410 nm, in water (gray) and just after addition to a suspension of PMVEMA-Es NPs (blue). Inset: Evolution of the fluorescence intensity of HTMA-PFP measured at 410 nm 20 h after its addition to the NP suspension. $\lambda_{exc}\;$= 380 nm.

Two strategies for the preparation of blue-emitting NPs were considered: either the polyfluorene HTMA-PFP was added to the ethanolic solution of PMVEMA-Es during the fabrication process, or it was added to the suspension once the PMVEMA-Es NPs were obtained, as is described in Materials and Methods. After fabrication of the fluorescent NPs by both procedures, their fluorescence spectrum and size were characterized. For the two approaches, it was concluded that polyfluorene is incorporated in the nanoparticles, given the considerable increase in fluorescence intensity and the blue shift observed in its spectrum, with respect to what occurs in water (Figure 2A). However, a lower fluorescence signal was obtained when HTMA-PFP was added during the preparation process, as well as an increase in the hydrodynamic diameter of the final nanoparticle, from 176.4 ± 2.2 nm (PDI = 0.11) to 221.2 ± 6.2 nm (PDI = 0.12). In view of these results, we decided to prepare the fluorescent NPs by incorporating the polyfluorene once the PMVEMA-Es NPs were formed.

Once the methodology was selected, the incorporation kinetics, as well as the optimal concentration of polyfluorene to be incorporated in the nanoparticles, were explored. The kinetics of incorporation was followed by measuring the fluorescence intensity of HTMA-PFP (1.5 µM in terms of repeat units) just after addition to a solution containing PMVEMA-Es NPs (0.03 M) and compared to that observed when the same amount of HTMA-PFP was added to water. Figure 2B shows how, in the absence of NPs, the fluorescence intensity of HTMA-PFP, measured at 410 nm, was very low, decreasing even slightly with time; whereas, in the presence of NPs, the intensity increased abruptly as soon as it was added, and then continued to increase very slightly until it stabilized after several hours. This result suggests a two-step mechanism for the incorporation of the polyfluorene into the nanoparticles, as was previously observed for anionic liposomes [34,36]. The fast kinetics and large increase in fluorescence intensity indicate that probably the nature of the interaction between HTMA-PFP and PMVEMA-Es NPs is initially electrostatic and occurs between the quaternary amine groups of HTMA-PFP and the partially deprotonated carboxyl groups on the NPs surface. As a result of the interaction, the polyfluorene chains become more extended than in water, reducing the probability of aggregation and thus strongly increasing their fluorescence quantum yield. The slower kinetics suggest that after the interaction, hydrophobic forces contribute to the solubilization of HTMA-PFP, resulting in the insertion of the polyfluorene chains between the compact PMVEMA-Es chains. The fact that the fluorescence intensity remained stable 20 h after incorporation of HTMA-PFP into the NPs led us to believe that, once inserted, it does not change its location (inset in Figure 2B).

To estimate the optimal concentration of polyfluorene to be incorporated in the NPs, increasing concentrations of HTMA-PFP, up to 9.5 µM (in terms of repeat units), were added to a suspension of PMVEMA-Es NPs (0.03 M) and fluorescence spectra were recorded on the first and the fifth day after preparation. The results shown in Figure S5 indicate that, as expected, the fluorescence intensity increased with increasing polyfluorene concentration. However, 5 days after the preparation, the only sample able to maintain the initial fluorescence signal was the one corresponding to 1.5 µM of HTMA-PFP. This result suggests that probably for concentrations above 1.5 µM, part of the inserted polyfluorene chains gradually leaves the NPs, forming aggregates in water. Therefore, the concentration of 1.5 µM was selected as the most suitable for preparing fluorescent NPs.

The affinity of HTMA-PFP to the PMVEMA-Es NPs was also estimated in order to better characterize the interaction between both systems. To this end, the fluorescence spectra of the polyfluorene were recorded at increasing amounts of NPs (Figure S6). The experiment was carried out by preparing different NPs suspensions in water, with PMVEMA-Es concentrations ranging from 0 to 0.03 M, and adding the same concentration of HTMA-PFP (1.5 μM) to all of the samples. As was expected, an enhancement of the fluorescence intensity and a blue-shift in the spectrum was observed as increasing concentrations of PMVEMA-Es, to reach practically its maximum value at ~0.03 M. The inset in Figure S6 represents the increase in fluorescence, measured at the maximum of the spectrum, versus the concentration of PMVEMA-Es. This type of plot allows, by fitting the curve to Equation (2) (Materials and Methods), the determination of the partition coefficient of the HTMA-PFP between the PMVEMA-Es NPs and the aqueous medium, $K_{p}$, but only if the molar volume of the PMVEMA-Es in the nanoparticle is known. However, as described in Materials and Methods, since this value was not available, an apparent affinity constant $K_{a}=K_{p}\gamma =341\pm 34\;M^{-1}$ was estimated for the binding process (Table 2). From the fit of the plot it was also possible to conclude that for NPs composed of 0.03 M of PMVEMA-Es and 1.5 µM of HTMA-PFP, most of the polyfluorene (close to 95%) is bound to the nanoparticles.

**Figure 3:**
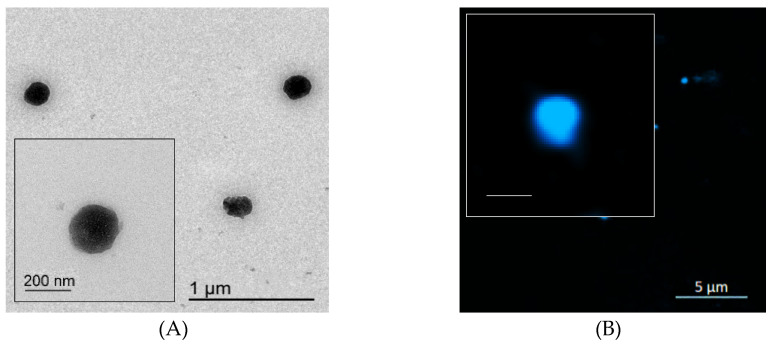
(**A**) Transmission electron microscopy (TEM) images of blue fluorescent NPs. (**B**) Fluorescence confocal microscopy images of blue fluorescent NPs.

The fluorescent NPs were also characterized by DLS and TEM to test whether the incorporation of polyfluorene into the nanoparticles affected their size or shape (Table 1 and Figure 3A). The obtained results show that the hydrodynamic diameter, PDI and morphology are quite similar to those corresponding to PMVEMA-Es NPs in the absence of HTMA-PFP, probably because most of the polyfluorene chains are embedded in the nanoparticle rather than coating the surface.

**Figure 4:**
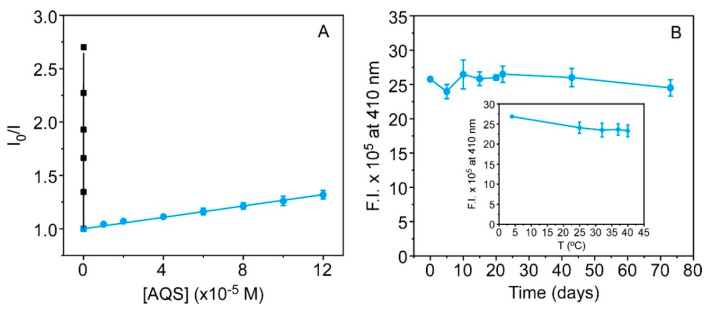
(**A**) Stern–Volmer plots for quenching of HTMA-PFP (1.5 µM) by AQS in MilliQ water (black squares) and in NPs (blue circles). (**B**) Fluorescence intensity, measured at 410 nm, of blue-emitting nanoparticles as a function of storage time. Inset: Fluorescence intensity of blue-emitting nanoparticles as a function of temperature. λ_exc_ =380 nm.

The above hypothesis regarding the location of HTMA-PFP in the NPs was tested by quenching experiments using the quinone-based electron acceptor AQS, an excellent quencher for cationic polyfluorenes, which is highly soluble in water but not in hydrophobic environments. The quenching efficiency was explored in a suspension containing the fluorescent NPs and compared to that obtained for HTMA-PFP in water. The results showed that the higher the concentrations of AQS, the lower the fluorescence intensity and this decrease was much more pronounced in water than in the NP suspension. Figure 4A shows the Stern–Volmer plots corresponding to these experiments. The plots were linear over the concentration range explored but the quenching efficiencies were clearly different in both systems. From the slope of the plots, using Equation (3) described in Materials and Methods, $K_{SV}$ values of 2.98 ± 0.05 × 10^7^ and 2.67 ± 0.05 × 10^3^ were obtained for polyfluorene in water and in the NP suspension, respectively (Table 2). The fact that the $K_{SV}$ value is much lower when HTMA-PFP is bound to the PMVEMA-Es NPs indicates that AQS can hardly access it and thus supports the hypothesis that most of the polyfluorene chains are embedded in the nanoparticle rather than coating its surface. This may be the reason why the ZP value of the PMVEMA-Es NPs is not affected after the incorporation of HTMA-PFP (Table 1).

The blue fluorescence of single nanoparticles was directly observed via super-resolution confocal microscopy and their diameters were measured, obtaining values comparable to those determined by DLS and TEM (Figure 3B). Moreover, the stability of their fluorescence signal was explored by monitoring the fluorescence intensity of HTMA-PFP, measured at 410 nm, as a function of storage time at 4 °C (up to 70 days) and temperature, showing that changes in the studied ranges, hardly modified the fluorescence of the NPs (Figure 4B). The slight decrease in fluorescence intensity with temperature increase is mainly due to the increased probability of non-radiative transitions, and not to temperature-induced aggregation of polyfluorene, since the initial fluorescence intensity was recovered after cooling.

**Figure 5:**
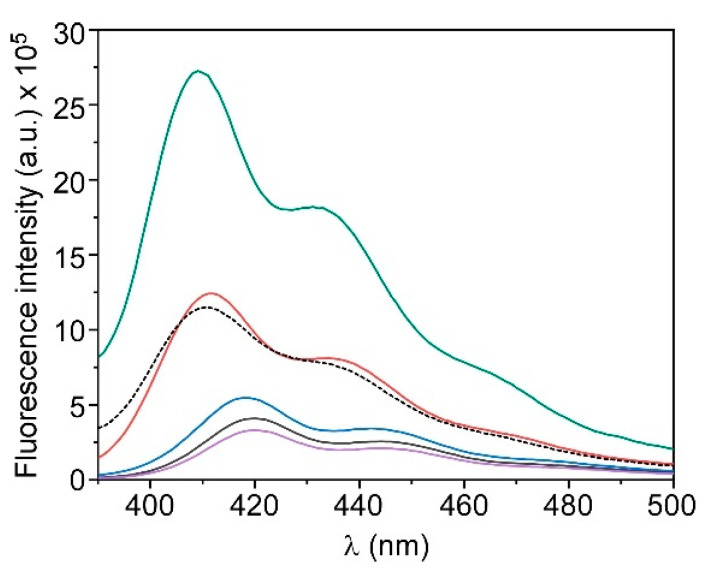
Alkalinisation effect on the fluorescence emission spectrum of blue fluorescent NPs at pH = 4 (green), pH = 5 (red), pH = 6 (blue), pH = 7 (black) and pH = 8 (purple). The dotted curve represents the emission spectrum of the NPs once the pH was reduced from 8 to 4 (λ_exc_ = 380 nm).

To find out whether the nanogel behavior of the NPs is maintained after the incorporation of the polyfluorene, the effect of pH on the size, PDI and ZP of the fluorescent NPs (Figure 1A,B,D), as well as the effect of ionic strength (Figure S4), were explored. The results showed a similar pattern to that observed in the absence of HTMA-PFP. In addition, fluorescent NPs also underwent reversible swelling-shrinking behavior as the pH decreased from pH 9 to acidic values, as shown in Figure S3. All these results provide evidence that the fluorescent NPs still exhibit characteristic properties of nanogels. An interesting fact was that the fluorescence intensity of the NPs decreased drastically with increasing pH, and the emission spectrum shifted to the red (Figure 5). This result suggests that the swelling of the nanogel, with the increase in water content, induces the aggregation of the polyfluorene chains, giving rise to enhanced interchain energy transfer, which in turn results in loss of fluorescence. When the pH was lowered again, the initial position of the spectrum was recovered, but not all the fluorescence signal, indicating that the process was not completely reversible and that part of the polyfluorene chains continue to aggregate. These aggregates probably remained inside the PMVEMA-Es NPs, since although the fluorescence was not fully restored, the size and polydispersity were recovered (Figure S3).

#### 2.2.2. Formation of Green and Red-Emitting NPs

**Figure 6:**
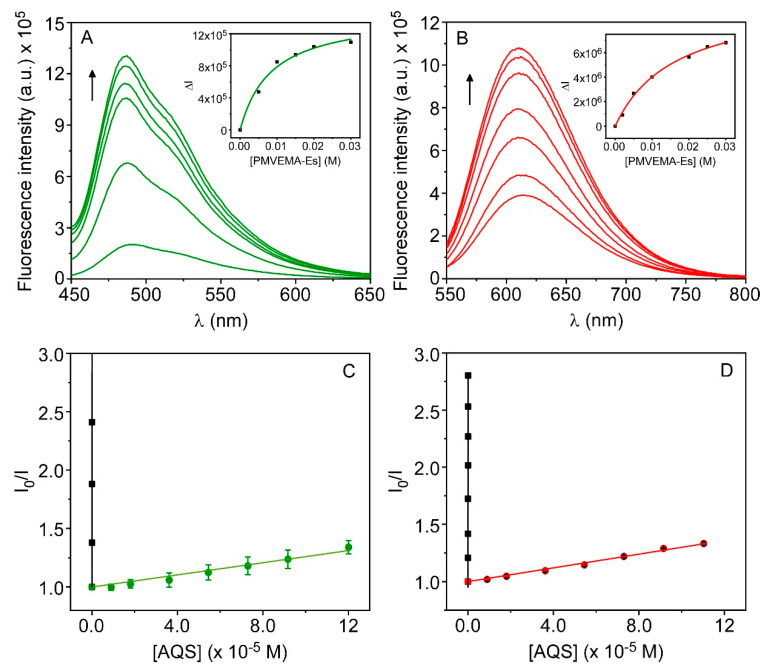
Fluorescence emission spectra of (**A**) HTMA-PFBT (1.5 µM) (λ_exc_ = 425 nm) and (**B**) HTMA-PFNT (1.5 µM) (λ_exc_ = 510 nm) in MilliQ water with increasing concentrations of NPs (0–0.03 M). Insets: Changes in the fluorescence intensity of the spectrum as a function of PMVEMA-Es concentration. Stern–Volmer plots for quenching of (**C**) HTMA-PFBT and (**D**) HTMA-PFNT (1.5 µM) in MilliQ water (squares) and in NPs (30 mM) (circles).

Fluorescent NPs with emission in the green and red regions were prepared with HTMA-PFBT and HTMA-PFNT, respectively, following the protocol optimized for blue-emitting NPs. The incorporation of both polyfluorenes into the PMVEMA-Es NPs was monitored through changes in their fluorescence. Figure 6A,B show, respectively, the emission spectra of HTMA-PFBT and HTMA-PFNT in samples containing increasing amounts of PMVEMA-Es. As in the case of HTMA-PFP, a low fluorescence emission signal was detected for the polyelectrolytes in water, while the presence of the NPs induced an increase in fluorescence intensity and a small blue shift of their emission spectra, confirming that both polyfluorenes bind to the polymeric NPs. Plotting the increase in intensity versus PMVEMA-Es concentration (inset in Figure 6) and fitting the data to Equation (2), apparent constants of $K_{a}$ = 119 ± 26 M^−1^ and $K_{a}$ = 62 ± 7 M^−1^ were obtained for HTMA-PFBT and HTMA-PFNT, respectively (Table 2), indicating that both polyfluorenes, especially HTMA-PFNT, show less affinity for polymeric NPs than HTMA-PFP, probably because they present a more tendency to aggregate in water.

The size and stability of green and red-emitting NPs, composed of 0.03 M of PMVEMA-Es and 1.5 µM of polyfluorene, was explored by DLS and fluorescence measurements as a function of storage time at 4 °C (Figure S7). The results show that the size of the NPs was similar to that determined for the blue-emitting NPs and it was maintained over time. In contrast, the fluorescence intensity was only preserved for the green-emitting NPs, while a continued decrease was observed for the red ones. This behavior suggests that HTMA-PFNT, despite initially incorporating into the polymeric NPs, gradually leaves them, forming aggregates in water, probably due to its higher hydrophobic character, compared to that of HTMA-PFBT and especially to that of HTMA-PFP [35]. To overcome this drawback, we tried to prepare the red-emitting NPs by adding the HTMA-PFNT to the ethanolic solution of PMVEMA-Es during the fabrication process, instead of incorporating it afterwards. Figure S7 shows the stability study performed at 4 °C for this new preparation. Although the intensity of the fluorescent NPs was lower than that obtained with the previous procedure, and the hydrodynamic diameter slightly higher, the most important finding was that both the fluorescence intensity and the size of the nanoparticles were preserved over time.

Additional characterization was made on the green and red fluorescent NPs. Table 1 shows that the ZP values were similar to those obtained for the blue-emitting NPs, confirming their colloidal stability and suggesting the insertion of the polyfluorenes in the polymeric NPs. To further investigate this assumption, their final localization was explored by AQS quenching experiments. A strong decrease in fluorescence was observed as increasing concentrations of the quencher were added to samples containing either HTMA-PFBT or HTMA-PFNT in water (1.5 µM). In contrast, when the same experiment was performed in samples containing green and red fluorescent NPs, the quenching effect was much less efficient. The Stern–Volmer plots were linear in all the studied ranges (Figure 6C,D) and the obtained Ksv values are shown in Table 2. As observed for HTMA-PFP, results support the hypothesis that HTMA-PFBT and HTMA-PFNT are incorporated within PMVEMA-Es NPs and are poorly accessible to the quencher.

**Figure 7:**
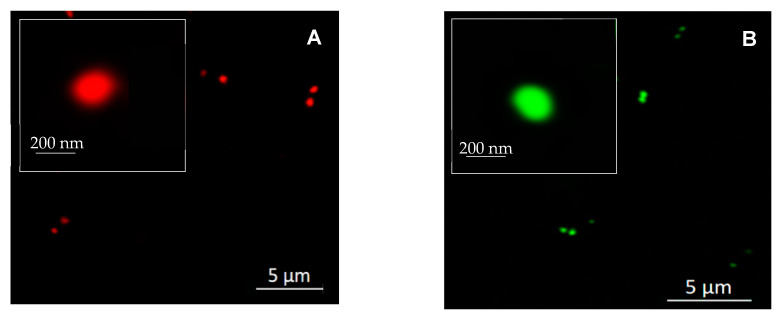
Fluorescence confocal microscopy images of (**A**) red- and (**B**) green-emitting NPs.

The green and red fluorescence of single NPs was observed by super-resolution confocal microscopy as is shown in Figure 7. Results indicate that, as blue-emitting NPs, they are rather spherical, with a size in agreement with those obtained from DLS experiments.

Finally, to show that the green and red fluorescent NPs still behaved as nanogels, their size and PDI were measured by DLS, first in water, then alkalinizing the sample to pH 7 and finally after acidification at pH 4 (Table S1). As expected, the hydrodynamic diameter and PDI of the NPs increased at physiological pH, especially in the case of red-emitting NPs, while decreasing again with decreasing pH, thus confirming the swelling/shrinking behavior of the fluorescent NPs in response to pH.

### 2.3. Fluorescent Nanogels as Drug Carriers and Image Probes

#### Ability of Blue-Emitting NPs to Bind DOX

Once the fluorescent NPs were prepared, characterized and tested for stability in water and acidic media, their ability to bind and transport drugs was explored. For this study, blue-emitting NPs were used as carriers and the antitumor drug DOX as a model drug. DOX was selected because it is fluorescent and its emission band does not interfere with that of HTMA-PFP, thus facilitating the study of the interaction between the two systems.

**Figure 8:**
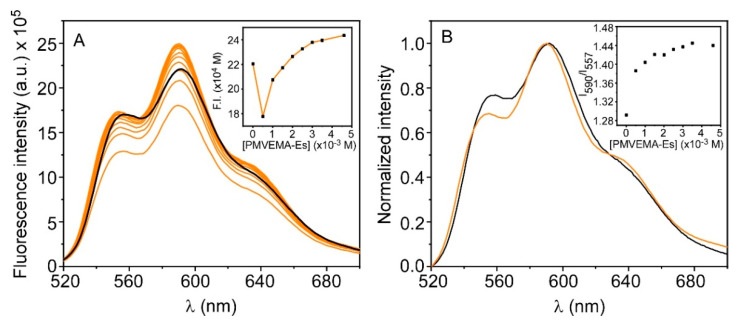
(**A**) Fluorescence emission spectra of DOX in water (black) and at increasing amounts of blue-fluorescent NPs (orange). Inset: Fluorescence intensity of DOX recorded at 590 nm, at increasing concentration of NPs. (**B**) Normalized spectra corresponding to DOX in water (black) and in the presence of NPs, at the maximum concentration added (orange). Inset: change in the ratio of the intensities of DOX emission peaks, R = I_590_/I_557_, versus [PMVEMA-Es]/[DOX] (λ_exc_ = 480 nm).

The first experiments focused on determining whether there is the interaction between the fluorescent NPs and DOX and, if so, estimating the affinity between the two components and exploring the nature of such interaction. For this purpose, NPs of PMVEMA-Es (0.03 M) and HTMA-PFP (1.5 µM) were prepared in water, and aliquots of this suspension were added to a water solution of DOX (1.25 µM), recording the emission spectra of the drug after each addition. DOX presents a fluorescence emission spectrum with two well-pronounced peaks at 557 and 590 nm, the latter being normally the one with the highest intensity, and a shoulder around 640 nm. The ratio of intensities of both peaks depends on the dielectric constant of the solvent in which it is found [51], this is why this feature can be used to assess whether DOX is able to be incorporated into NPs. The fluorescence intensity also is sensitive to the environment, however, this property is less reliable to quantify the binding capacity, since other factors, such as dimer formation, self-quenching and reabsorption phenomena also contribute to modify it [52]. Figure 8A shows the emission spectra of DOX recorded at the increasing amounts of NPs. The spectrum in the absence of PMVEMA-Es exhibited a good fluorescence intensity, the first addition sharply decreased this signal and then successive additions increased the fluorescence again. This behavior is best observed in the inset of Figure 8A, where the fluorescence intensity of DOX at 590 nm is plotted as a function of PMVEMA-Es concentration. This result could be explained by the previously mentioned aggregation and “self-quenching” capacity of DOX, which occurs when the molecules are in close proximity to each other. After the first addition, a large part of the DOX molecules that were in the water are incorporated into the NPs, so that the local drug concentration increases enormously, leading to a sharp decrease in fluorescence intensity. Successive additions of NPs “dilute” the drug concentration, so the signal increases again. Therefore, the changes in fluorescence intensity suggest that DOX interacts with the NPs, but cannot be used to quantify such interaction.

To better observe the effect of the fluorescent NPs on the DOX emission spectrum, the normalized spectra corresponding to the drug in water and in presence of NPs, at the maximum concentration added, are shown in Figure 8B. In them, it can be seen how the presence of NPs induces a slight blue shift of the DOX spectrum, as well as a clear change in the ratio of the emission peak intensities, i.e., R = I_590_/I_557_. This ratio gradually increased from 1.29 to ~1.46 as the PMVEMA-Es concentration was increased, reaching a plateau at ~0.003 M (Inset in Figure 8B). According to the literature, this result confirms that DOX interacts with the NPs and localizes in an environment with a lower dielectric constant than water, but not totally hydrophobic [51], suggesting that the drug remains relatively close to the NPs surface. This presumable location of DOX in the NPs could be explained by taking into account the pK_a_ of the primary amine group in DOX, which is 8.2. Because our experiments are conducted in water, the amino carbohydrate moiety of DOX would be expected to be mostly positively charged, interacting electrostatically with the anionic surface of the NPs and thus preventing deep penetration into their interior [51,53].

The above results also allow the estimation of the affinity of the drug for the blue-emitting NPs. To this end, the changes in the ratio of emission peaks, ΔR = (I_590_/I_557_) − (I_590_/I_557_) water, were plotted in a graph versus PMVEMA-Es concentration (Figure S8) and fitted to Equation (2), previously modified by replacing ΔI and ΔI_max_ with ΔR and ΔR_max_, respectively. From this fit, it was possible to determine an apparent affinity constant *K_a_* = 2471 ± 326 M^−1^ for the binding process. A similar experiment was carried out, but with PMVEMA-Es NPs in the absence of polyfluorene (Figure S8). The obtained affinity constant *K_a_* = 1754 ± 242 M^−1^ was only slightly lower than the previous one, suggesting that HTMA-PFP has very little influence on drug binding to the NPs.

**Figure 9:**
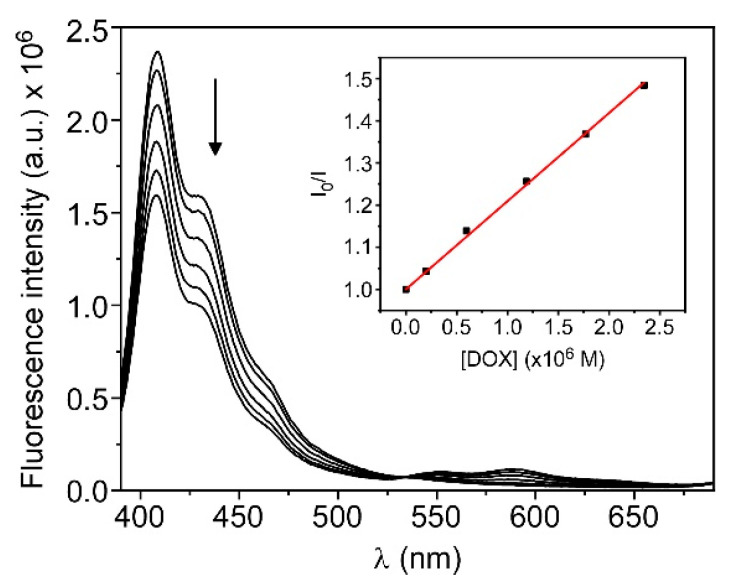
Fluorescence emission spectra of blue-emitting NPs at increasing concentrations of DOX (0–24 µM). λ_exc_ = 350 nm. Inset: Stern–Vomer plots for quenching of NPs by DOX.

The interaction of DOX with blue-emitting NPs was also monitored from changes in the fluorescence properties of the NPs, instead of those of the drug, taking advantage of the fact that the emission bands of both, DOX and HTMA-PFP, are well separated. To this end, increasing concentrations of DOX (0–24 µM) were added to a suspension of fluorescent NPs and their emission spectra were recorded at each addition. Figure 9 displays the spectra obtained after correction for the inner-filter effect, using Equation (6). Results show that as the DOX concentration increases, the intensity of the emission maximum of NPs gradually decreases, whereas the spectrum is not shifted. As for AQS, the quenching behavior can simply be described by the Stern–Volmer equation (Equation (3)). A plot of I_0_/I versus the concentration of DOX is shown in the inset of Figure 9. A linear relationship is obtained which provides a *K_SV_* = 2.1 × 10^4^ M^−1^. It is known that fluorescence quenching in polyfuorenes mainly arises from energy transfer (FRET) and/or photoinduced electron transfer (PET) between donor and acceptor [54,55]. Efficient FRET usually requires good spectra overlap between donor emission and acceptor absorption spectra. For DOX, its absorption spectrum partially overlaps with the emission spectrum of the fluorescent NPs (Figure S9), so the quenching of the NPs could be in principle attributed to this mechanism. To explore this hypothesis, the Förster radius (*R_o_*) was determined from Equations (4) and (5), described in Materials and Methods. The quantum yield of the donor (the polymer inserted in the NPs) necessary to carry out this calculation was determined using quinine sulfate as a reference, obtaining a value of Φ*_F_* = 0.84. From the spectral overlap shown in Figure S9, the overlap integral J for the system was found to be 1.9 × 10^14^ cm^−3^ M^−1^. Considering these parameters, a *R_0_* = 36.14 Å was determined which supports the possibility of the FRET mechanism is responsible for the quenching of the fluorescent NPs. Such mechanism, in which DOX acts as acceptor, is not uncommon and has been described on other occasions for the binding of DOX to fluorescent materials [56]. However, if this were the predominant mechanism, increasing the DOX content would not only gradually decrease the fluorescence intensity of the nanoparticles, but should also gradually increase the fluorescence intensity of the DOX upon exciting the HTMA-PFP. Note that the NPs were excited at 350 nm instead of 380 nm to minimize DOX absorption. Although Figure 9 shows that the quenching of NPs is followed by a slight increase in fluorescence of DOX, we found that the same emission bands were obtained when the blank (DOX added to NPs in absence of HTMA-PFP) were recorded under the same conditions, as a consequence of the still small absorption of the drug at 350 nm. This result implies that FRET is not the dominant quenching mechanism and suggests that PET is the more likely mechanism. The fact that DOX contains a quinone group in its structure, which is a very efficient electron transfer quencher (note that AQS also contains this group), supports this hypothesis. In addition, other works have also reported that DOX is a potent electron acceptor involved in the quenching of fluorophores through the PET process [57,58,59]. Since this mechanism only operates over very short distances, this would indicate that the DOX molecules have to be sufficiently close to the HTMA-PFP backbone or in Van der Waals contact to induce the quenching.

**Figure 10:**
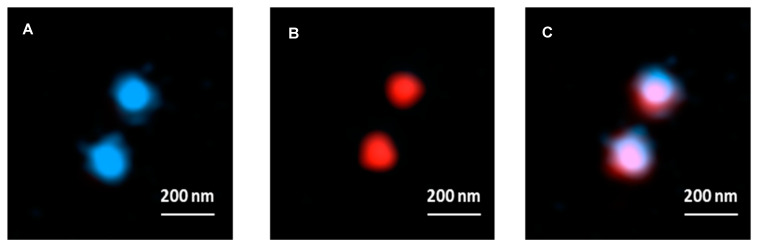
Confocal fluorescence microscopy images of blue-emitting NPs containing DOX (20 µM) after being excited at 405 nm (**A**), 509 nm (**B**) and the overlapping of both wavelenghts (**C**).

The blue and red fluorescence emitted by individual nanogels due to the presence of HTMA-PFP and DOX, respectively, could be observed by super-resolution confocal microscopy. The images in Figure 10 show the same NPs after being excited with UV (Figure 10A) and Vis light (Figure 10B), while Figure 10C displays the overlapping of both images, demonstrating that both components coexist in the same NP. The fact that the blue fluorescence of the NPs is still observed despite the presence of DOX confirms that the drug only quenches the polyfluorene chains with which it comes in contact and not the rest of the polyfluorene incorporated in the NPs.

To demonstrate the stability of the nanoformulations in water, the fluorescence spectra of the drug were recorded under different conditions. Inset in Figure S10 shows that the increase in temperature from room to physiological temperature, kept the drug inserted in the NPs, since the emission peak ratio, R, was preserved. Similarly, it was also proved that DOX remained bound to the nanoparticles for at least 12 days, at 4 °C, since R also did not change during this period of time (Figure S10).

**Figure 11:**
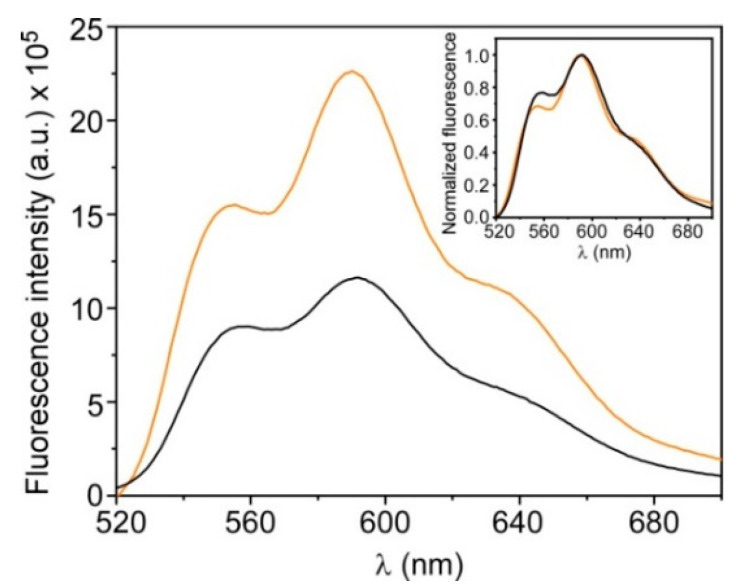
Fluorescence emission spectra of DOX incorporated in NPs before (orange) and after bringing the suspension to physiological pH (black). Inset: Normalized spectra corresponding to Figure 11.

Furthermore, considering the nanogel-like behavior of the NPs, we explored whether the increase in pH induced the drug release as a consequence of the swelling effect. To this end, fluorescent NPs containing DOX (20 µM) were prepared in water and subsequently were brought to physiological pH. Figure 11 displays the fluorescence spectrum of DOX before and just after the pH change. The results show that at physiological pH the fluorescence intensity of DOX decreased and the ratio of the emission peaks intensities recovered the value corresponding to free DOX (inset in Figure 11), evidencing that the swelling of the NPs induces the release of the DOX.

**Scheme 2:**
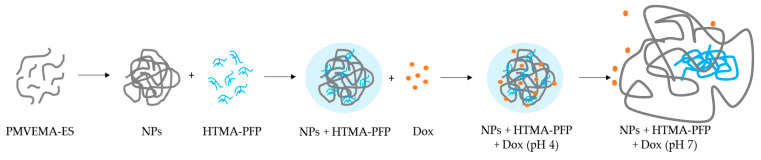
Schematic model which shows the formation of blue-emitting NPs containing DOX and the drug release as a consequence of the pH-induced swelling effect.

As a summary, Scheme 2 shows the preparation of the fluorescent nanoformulations and the subsequent release of the drug as a consequence of the pH change.

### 2.4. NPs as Image Probes

**Figure 12:**
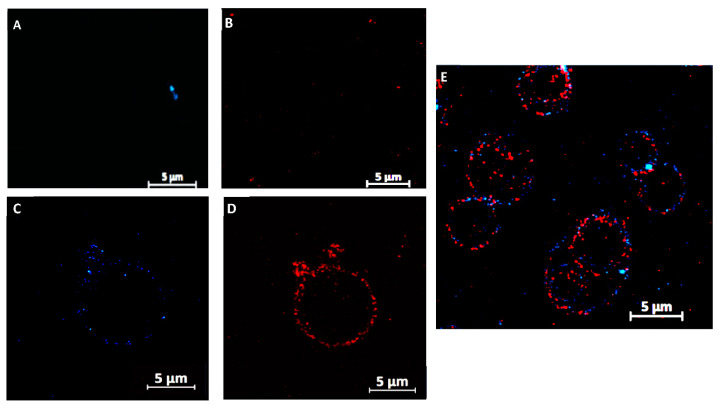
Confocal microscopy images of GUVs of DOPC in presence of fluorescent nanogels containing DOX, upon excitation at 410 nm (**A**,**C**) and 509 nm (**B**,**D**) in water (**A**,**B**) and at pH 7 (**C**,**D**). Image showing the overlap of the blue and red emission of a population of GUVs after being excited with UV and Vis light, in the presence of the nanogels at pH 7 (**E**).

Finally, we performed preliminary experiments to explore the ability of the fluorescent nanogels to provide imaging functions. To this end, giant unilamellar vesicles (GUVs) which mimic the plasma membrane of mammalian cells were prepared in glucose 0.2 M as is described in Methods. Once prepared, they were incubated with blue-emitting NPs containing DOX 20 µM, and observed under the confocal microscope. When samples were excited with UV and Vis light, Figure 12A,B, respectively, no labeling of GUVs was observed, neither in the blue region, characteristic of HTMA-PFP, nor in the red region, characteristic of DOX, and only some blue and red fluorescent points could be visualized.

This result suggests that under these conditions, the NPs do not bind to the cell membrane probably because of their high stability in water and glucose, which was shown in the previous sections. However, when an aliquot of buffer (pH 7) was added to the samples, the GUVs could be clearly visualized. This can be seen in Figure 12C,D whose images correspond to the same field. When NPs were excited with UV light, GUVs were labeled in blue (12C), while when exciting with Vis light, they were labeled in red (12D). Figure 12E shows another image in which the blue and red emission of a population of GUVs are overlapped after being excited with UV and Vis light, in the presence of the NPs and at physiological pH. These results indicate on the one hand that the swelling of the NPs, caused by the increase in pH, induces the release of DOX molecules and these are incorporated into the lipid membrane of the GUVs. On the other hand, the polyfluorene also binds to the vesicles, given its high affinity for this medium, acting as a membrane marker and thus signaling that the nanoparticle has released its contents.

## 3. Materials and Methods

### 3.1. Materials and Reagents

The copolymer PMVEMA-Es (M_w_~130 kg/mol), provided as 50% *w*/*w* solution in ethanol, and the antitumor drug DOX (M_w_ = 579.98 g/mol) were purchased from Merck Life Science (Madrid, Spain). The last was dissolved in DMSO (1 mM) while PMVEMA-Es was used as received. The cationic CPEs HTMA-PFP (M_n_ = 4170 g/mol; M_w_ = 8340 g/mol), HTMA-PFBT (M_n_ = 4584 g/mol; M_w_ = 8531 g/mol) and HTMA-PFNT (M_n_ = 4507 g/mol; M_w_ = 8990 g/mol) were previously prepared in our laboratory and dissolved in DMSO (3.65 × 10^−4^ M, 6.24 × 10^−4^ M and 3.65 × 10^−4^ M in terms of repeating units, respectively) [35,36,38]. The quencher 9,10-anthraquinone-2,6-disulfonic acid (AQS) (M_w_ = 208.21 g/mol) was purchased from Merck Life Science (Madrid, Spain) and prepared as 5 mM stock solution in twice distilled and deionized water, utilizing Milli-Q equipment (Millipore, Madrid, Spain). The compounds used for giant unilamellar vesicle (GUVs) experiments—synthetic phospholipid 1,2-dioleoyl-sn-glycero-3-phosphocholine (DOPC) (M_w_ = 786.11 g/mol), glucose 0.2 M (M_w_ = 180.16 g/mol) and sacarose 0.2 M (M_w_ = 342.30 g/mol)—were also purchased from Merck Life Science (Madrid, Spain). The phosphate buffer (50 mM, 0.1 M NaCl, pH 7.4) was prepared in MilliQ water. The rest of compounds were of analytical or spectroscopic grade.

### 3.2. Preparation of PMVEMA-Es Nanoparticles (PMVEMA-Es NPs)

Polymeric NPs were prepared by slightly modifying the solvent displacement method previously described in the bibliography [60]. Briefly, different amounts of the ethanolic solution of PMVEMA-Es (from 10 to 40 mg) were dissolved in 1.25 mL of ethanol. The suspension was incubated for 10 min at room temperature under magnetic stirring. Straightaway, 250 µL of MilliQ water were added dropwise. After 10 min of incubation with magnetic stirring, another 1.25 mL of MilliQ water were added dropwise, and the mixture was left stirring for another 10 min. Finally, the organic solvent was removed by vacuum rotary-evaporation, and the volume was completed up to 2.5 mL with MilliQ water, obtaining final PMVEMA-Es concentrations ranging from 10 to 40 mM, in terms of repeat units. The hydrodynamic diameter of NPs, measured by dynamic light scattering, increased from 100 to 325 nm with increasing polymer concentration (Figure S11), as was previously observed by members of our group [13]. According to the literature, nanoparticles <10 nm tend to be more easily excreted, while nanoparticles >200 nm tend to experiment nonspecific interactions with biological components, inducing their opsonization and subsequent elimination [61]. Nevertheless, as a rule, the greater amount of polymer, the greater amount of drug entrapped, so the PMVEMA-Es concentration of 30 mM was chosen for most of the experiments performed in this work.

### 3.3. Preparation of Multicolor Fluorescent PMVEMA-Es NPs

Blue fluorescent NPs were prepared by adding HTMA-PFP during or after the PMVEMA-Es NPs synthesis. If added during the process, HTMA-PFP was included once the PMVEMA-Es was dissolved in 1.25 mL of ethanol. Straightaway, the solution was incubated for 20 min at room temperature under magnetic stirring. Further steps were similar to the protocol previously described. If added after the process, HTMA-PFP was included by externally adding aliquots of the polyfluorene dissolved in DMSO to the polymeric NPs suspension. To ensure the complete incorporation of HTMA-PFP inside the polymeric nanoparticles, the suspension was incubated for 30 minutes at room temperature. The final HTMA-PFP concentration was 1.5 µM and the proportion of DMSO was always lower than 1% *v*/*v*. Green and red fluorescent PMVEMA-Es NPs as the blue ones but replacing HTMA-PFP by HTMA-PFBT and HTMA-PFNT, respectively.

### 3.4. Incorporation of DOX in the Fluorescent NPs

Fluorescent NPs containing DOX were prepared by externally adding aliquots of the drug dissolved in DMSO to the polymeric nanoparticle suspension. To ensure the incorporation of the DOX in the polymeric NPs, the final suspension was incubated for 30 min at room temperature. In all the cases, the proportion of DMSO was always lower than 1% *v*/*v*.

### 3.5. Preparation of GUVs

Giant unilamellar vesicles (GUVs) were prepared by electroformation method. Briefly, 10 µL of DOPC (1 mM) dissolved in CHCl_3_ was placed on the conductive side of an ITO-coated slide (Munich, DE). The sample was dried vacuum for 1 h 15 min at room temperature. Straightaway, 280 µL of 0.2 M sucrose were added. The mixture was covered with another ITO-slide and coupled to the Nan[i]on Vesicle Prep Pro (Munich, DE). An electroformation process of 10 Hz, 3 V, 37 °C and 188 min was carried out. Finally, 25 µL of the obtained vesicles were dissolved in 300 µL of 0.2 M glucose on a µ-Slide 8 Well IBIDI^®^ Cell in focus multi-well plate (Gräfelfing, DE).

### 3.6. Dynamic Light Scattering and Zeta Potential

Dynamic light scattering (DLS) was applied to measure the average hydrodynamic diameter and polydispersity of the nanoparticles. The analysis of Zeta Potential (ZP) was also carried out to examine the surface charge and colloidal stability of nanoparticles in solution. These measurements were performed using a Malvern ZetaSizer Nano-ZS instrument (Worcestershire, UK) equipped with a monochromatic coherent 4 mW Helium Neon laser (λ = 633 nm) light source, where size measurements were performed at angles of 173°. All of them were carried out in triplicate and at room temperature (~25 °C), using disposable cuvettes for size measurements and specific folded capillary cells for Zeta Potential measurements. Data in the corresponding graphs represent the mean of the measurements, with error bars corresponding to the standard error of the mean.

### 3.7. pH Titration

The pH titrations were conducted in a Malvern ZetaSizer Nano-ZS equipment (Worcestershire, UK) equipped with an MPT-2 pH titrator. The pH was modified with HCl 1 M and NaOH 1 M solutions. Measurements were carried out in triplicate and at room temperature (~25 °C), using specific folded capillary cells. Data in the corresponding graphs represent the mean of the measurements, with error bars corresponding to the standard error of the mean.

### 3.8. Fluorescence Microscopy

Images of the blue, green and red fluorescence of the nanoparticles were acquired with a laser scanning confocal microscope Zeiss LSM 900 (Berlin, DE) equipped with an Airyscan 2 super-resolution detector and processed using Zeiss ZEN 3.2 (Blue Edition) software. The recording was acquired with a 63×/1.4NA objective (oil-immersion) and the filters DAPI (Ex. 405 nm), Alexa Fluor 488 (Ex. 488) and Alexa Fluor 509 (Ex. 509 nm). Images of GUVs were also acquired using the same microscope.

### 3.9. Electron Microscopy

Transmission electron microscopy (TEM) was performed by using a transmission electron microscope (JEM-1400 Plus, JEOL, Tokyo, Japan), working at 120 kV. Images of NPs were obtained by placing a drop of the corresponding suspension on 300-mesh copper grids coated with carbon from Electron Microscopy Science (Hatfield, PA, USA). These samples were left to dry before being placed under the microscope.

### 3.10. Fluorescence Experiments

A PTI-QuantaMaster spectrofluorimeter (PTI, Birmingham, NJ, USA) equipped with a Peltier cell to monitor temperature was used for fluorescence measurements. The samples were placed in quartz cuvettes (1 cm × 1 cm) and subsequently excited at 380 nm (HTMA-PFP), 425 nm (HTMA-PFBT), 510 nm (HTMA-PFNT) or 480 nm (DOX). Measurements were performed in triplicate and background noise was measured and subtracted whenever it was necessary. All experiments were carried out at 25 °C, except for those in which the effect of temperature was evaluated. Data in the corresponding graphs represent the mean of the measurements, with error bars corresponding to the standard error of the mean.

### 3.11. Affinity Measurements

The partition coefficient ($K_{p}$) of the CPEs and DOX between the NPs and the aqueous medium, $K_{p}$, is defined in terms of molar concentrations as:

(1) $$K_{p}=\frac{n_{PMVEMA}^{c}/V_{PMVEMA}}{n_{Aq}^{c}/V_{Aq}}$$

where $n_{i}^{c}\;$stands for moles of compound in phase *i* and *V_i_* for volume of phase *i*. The phase is either aqueous (*i* = *Aq*) or polymeric (*i* = *PMVEMA*). $K_{p}$ can be estimated from changes in some fluorescence parameter, such as fluorescence intensity of CEPs, in presence of increasing concentrations of *PMVEMA-Es*, according to Equation (2) [62]:

(2) ∆I=∆Imax[PMVEMA]1(Kpγ)+[PMVEMA]

In this Equation, $∆I\;\left(∆I=I-I_{0}\right)$ stands for the difference between the emission areas or fluorescence intensities of CPEs in the absence ($I_{0}$) and in the presence (I) of *PMVEMA-Es*; $∆I_{max\;}\;\left(∆I_{max}=I_{\infty }-I_{0}\right)$ represents the maximum value of this difference once the highest concentration of *PMVEMA* was added, and γ stands for the molar volume of *PMVEMA* in the nanoparticles. Since we do not know the value of γ, we could only determine an apparent affinity constant *K_a_* = $K_{p}\gamma$.

### 3.12. Fluorescence Quenching Experiments

To explore the location of polyfluorenes in the polymeric NPs, their fluorescence emissions were studied in the presence of different AQS concentrations. According to the literature, AQS is an electron acceptor which acts as a quencher of CPEs fluorescence [54,63]. Stern–Volmer studies were conducted according to Equation (3):

(3) $$I_{0}/I=1+K_{SV}\left[Q\right]$$

In this Equation, I and $I_{0}$ stand for the fluorescence intensities in the presence and in the absence of the quencher, [Q] represents the concentration of AQS and $K_{SV}$ the Stern–Volmer constant, whose value gives information on the efficiency of the quenching process and reflects the accessibility of the fluorophores to AQS.

The interaction of DOX with fluorescent NPs was also explored from quenching experiments in which DOX acted as a quencher of blue-emitting NPs.

### 3.13. Förster Energy Transfer Experiments (FRET)

In these experiments, the DOX was used as an acceptor of the HTMA-PFP excitation. FRET studies were conducted according to Equations (4) and (5), where $R_{0}$ represents the distance between the donor and acceptor at which the FRET efficiency is 50%:

(4) $$R_{0}=0.2108\;{\left[k^{2}\Phi_{F}n^{-4}J\right]}^{\left(1/6\right)}$$

(5) $$J={\int^{\;}}_{0}^{\infty }I\left(\lambda \right)\;\varepsilon \left(\lambda \right)\;\lambda^{4}\;d\lambda$$

In these Equations, $k^{2}$ represents the orientation factor between HTMA-PFP and DOX (2/3 for random orientations); $\Phi_{F}\;$is the quantum yield of the fluorescence of HTMA-PFP in the absence of DOX; n stands for the refractive index of the medium (1.425); I corresponds with the fluorescence intensity of HTMA-PFP; ε represents the molar extinction coefficient of DOX; and λ is the wavelength. The fluorescence emission spectra of HTMA-PFP in the presence of DOX was excited at 350 nm instead of 380 nm to minimize DOX absorption. The inner filter effect correction was also applied by using the equation described by Lakowicz (Equation (6)) [64]:

(6) $$I_{corr}=I_{obs}\;10^{\frac{\left(A_{\lambda exc}+A_{\lambda em}\right)}{2}\;}$$

In this Equation, $I_{corr}$ represents the fluorescence intensity after correction; $I_{obs}\;$ stands for the observed fluorescence intensity; $A_{\lambda exc}\;$ corresponds with the absorption of DOX at the excitation wavelength (350 nm) of HTMA-PFP; and $A_{\lambda em}\;$ is the absorption of the DOX at the emission wavelengths (390–500 nm) of HTMA-PFP.

## 4. Conclusions

In the present study, we developed and characterized blue, green and red fluorescent NPs composed of a polymeric core of PMVEMA-Es and the polyfluorenes HTMA-PFP, HTMA-PFBT and HTMA-PFNT, respectively, in order to obtain multicolor fluorescent drug carriers. The incorporation of the polyfluorenes in the PMVEMA-Es nanoparticles is very fast and probably takes place through electrostatic interactions between the quaternary amine groups of the polyfluorenes and the partially deprotonated carboxyl groups on the PMVEMA-Es core surface. After the interaction, hydrophobic forces seem to contribute to the solubilization of the polyfluorenes, resulting in the insertion of their chains between the compact PMVEMA-Es chains.

The obtained NPs are monodisperse in size and exhibited stable fluorescence signals, good colloidal stability, spherical morphology and diameters below 200 nm, when prepared and stored in milli-Q water or in acidic pH aqueous solutions. NPs are also stable with temperature, but exhibited pH-dependent response, swelling up to four times their size at physiological pH, and shrinking to their initial size at acidic pH. Such behaviour is attributed to the carboxylic acid group of PMVEMA-Es, whose pKa is ~5.3, and indicates that these fluorescent nanocarriers can be categorized as pH-sensitive nanogels.

DOX was used as a model drug to explore the ability of the developed nanogels to hold drugs in acidic media and release them at physiological pH. The binding was demonstrated by changes in the fluorescence spectra of DOX after incorporation into the nanoparticle, as well as by fluorescence quenching of the blue-emitting nanogels, most likely caused by a photoinduced electron transfer process between HTMA-PFP and DOX. Once the drug was bound, the nanoformulation could be visualized by confocal microscopy, and remained stable over time at 4 °C. However, the drug was immediately released when the pH of the medium was increased to pH 7, as a consequence of the swelling of the nanogel.

Finally, preliminary experiments with GUVs showed the capability of the fluorescent nanogels to mark and visualize membrane structures by fluorescence microscopy, but only when the nanogel adopts a swollen conformation, allowing the DOX molecules to access the biological membrane.

All these results suggest that the developed nanogels could be used to transport and protect sensitive drugs from the acidic conditions of the stomach, offering excellent potential as oral therapeutic systems. In this sense, to complete an oral drug delivery system, microencapsulation of the nanoparticles in suitable polymeric systems, that prevent degradation of the nanogels and premature release of the drug into the oral cavity and esophagus, is necessary. Therefore, the development of microcapsules and the encapsulation of nanogels in them will be one of the objectives of forthcoming work. We also plan to further expand the applications of the nanoparticles in the future by functionalizing the fluorescent nanogels with suitable ligands, in order to improve selectivity against specific targets and enable multiplexed bioimaging of cells.

## Figures and Tables

**Table 1:** Hydrodynamic diameter (d), polydispersity index (PDI) and Zeta Potential (ZP) of PMVEMA-Es NPs and blue, green and red fluorescent NPs in Milli-Q water.

	NPs	NPs + HTMA-PFP	NPs + HTMA-PFBT	NPs + HTMA-PFNT ^1^
d (nm)	171.3 ± 0.7	176.4 ± 2.2	172.0 ± 4.2	194.6 ± 4.0
PDI	0.12 ± 0.01	0.11 ± 0.02	0.12 ± 0.03	0.13 ± 0.02
ZP (mV)	−27.0 ± 1.0	−26.8 ± 0.8	−27.4 ± 0.6	−26.2 ± 0.5

^1^ added during the fabrication process.

**Table 2:** Apparent affinity constant ($K_{a}=K_{p}\gamma$) and Stern–Volmer constants ($K_{SV}$) for HTMA-PFP, HTMA-PFBT and HTMA-PFNT in NPs of PMVEMA-Es by using AQS as a quencher.

	$K_{a}\;\left(M^{-1}\right)$	$K_{SV}\;\left(M^{-1}\right)$
HTMA-PFP + NPs	341 ± 34	2.67 ± 0.05 × 10^3^
HTMA-PFP + MilliQ water		2.98 ± 0.05 × 10^7^
HTMA-PFBT + NPs	119± 26	2.60 ± 0.12 × 10^3^
HTMA-PFBT + MilliQ water		4.04 ± 0.17 × 10^7^
HTMA-PFNT + NPs	62 ± 7	3.00 ± 0.06 × 10^3^
HTMA-PFNT + MilliQ water		1.70 ± 0.24 × 10^6^

## Data Availability

The data presented in this study are available in the article and Supplementary Materials files.

## Supplementary Materials

The following are available online at https://www.mdpi.com/article/10.3390/ijms22179607/s1. Table S1: Effect of pH on the size and polydispersity index of green and red-emitting NPs; Figure S1: TEM microscopy of PMVEMA-Es NPs; Figure S2: Stability over time and with temperature of PMVEMA-Es NPs; Figure S3: Effect of pH on the size, polydispersity index and zeta potential of PMVEMA-Es NPs in absence and in presence of HTMA-PFP; Figure S4: Effect of NaCl on the size of PMVEMA-Es NPs; Figure S5: Fluorescence emission spectra of HTMA-PFP in PMVEMA-Es NPs at different concentrations of polyfluorene; Figure S6: Fluorescence emission spectra of HTMA-PFP with increasing concentrations of PMVEMA-Es NPs; Figure S7: Stability of green and red-emitting NPs; Figure S8: Affinity of the DOX for the PMVEMA-Es NPs; Figure S9: Efficient FRET between HTMA-PFP and DOX; Figure S10: Fluorescence stability of blue-emitting NPs containing DOX; Figure S11: Effect of PMVEMA-Es concentration on the size of PMVEMA-Es NPs.
