# Supplementary material for: Formation of Multicolor Nanogels Based on Cationic Polyfluorenes and Poly(methyl vinyl ether-alt-maleic monoethyl ester): Potential Use as pH-Responsive Fluorescent Drug Carriers
Source: Int J Mol Sci. 2021 Sep 4;22(17):9607. doi: 10.3390/ijms22179607 (PMC8431760; doi:10.3390/ijms22179607)
Supplement: Supplementary file 1 [file ijms-22-09607-s001.zip › ijms-1355155-supplementary.pdf]

Supplementary Material

Formation of Multicolor Nanogels Based on Cationic Polyfluorenes and Poly(methyl vinyl ether-alt-maleic monoethyl ester): Potential Use as pH-responsive Fluorescent Drug Carriers

Marta Rubio-Camacho [1], María José Martínez-Tomé [1,*], Amalia Mira [1], Ricardo Mallavia [1] and C. Reyes Mateo [1,*]

[1] Instituto de Investigación Desarrollo e Innovación en Biotecnología Sanitaria de Elche (IDiBE), Universidad Miguel Hernández de Elche (UMH), 03202 Elche, Alicante, Spain

Keywords: Nanogels; PMVEMA; fluorescent conjugated polymers; doxorubicin; nanoparticles; drug-delivery; bioimaging.

* Correspondence: mj.martínez@umh.es; rmateo@umh.es

Tabla S1. Effect of alkalinisation and subsequent acidification on the hydrodynamic diameter (d) and polydispersity index (PDI) of green and red-emitting NPs.

	medium	d ± sd (nm)	PDI
**Green NPs**	water	172.0 ± 4.2	0.12 ± 0.03
	pH 7	428.1 ± 28.6	0.25 ± 0.02
	pH 4	206.4 ± 6.0	0.13 ± 0.03
**Red NPs**	water	194.6 ± 4.0	0.13 ± 0.02
	pH 7	708.3 ± 11.4	0.27 ± 0.07
	pH 4	245.1 ± 8.5	0.18 ± 0.08

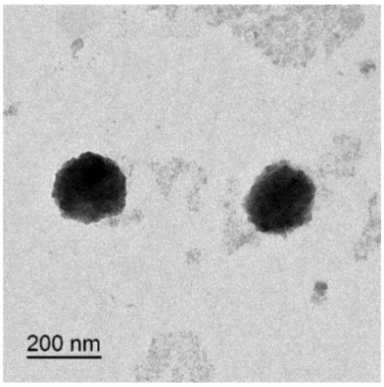

Figure S1. Representative images of transmission electron microscopy (TEM) of PMVEMA-Es NPs

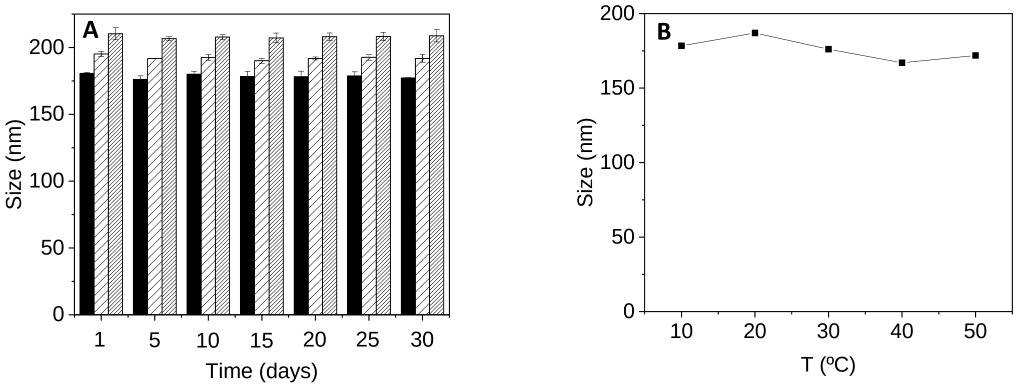

Figure S2. (**A**) Bar diagrams showing the hydrodynamic diameters of PMVEMA-Es NPs (0.03 M) as a function of time (1-30 days), in MilliQ water (black), glucose 0.2 M (striped) and sacarose 0.2 M (full striped). Note that the slight increase in size observed after addition may be related to the change in viscosity and refractive index of the medium, rather than a real increase in the size of the nanoparticles. (**B**) Effect of temperature (10-50ºC) on the hydrodynamic diameter of NPs (0.03 M) in MilliQ water.

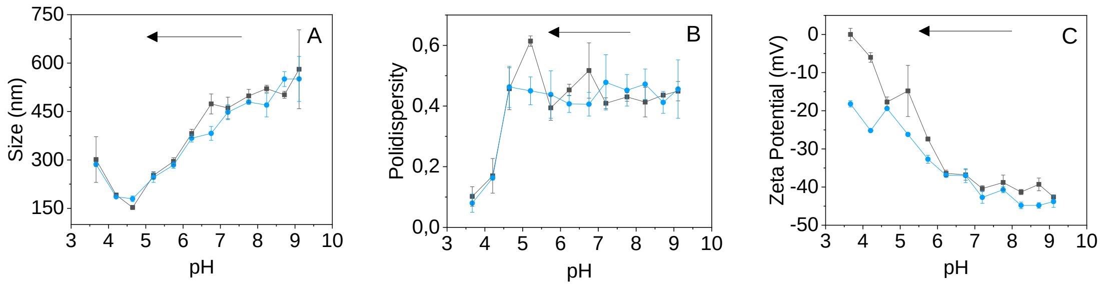

Figure S3. Reversible swelling–shrinking behaviour of PMEVEMA-Es NPs in absence (black) and in presence (blue) of HTMA-PFP, as the pH decreased from 9 to 3.

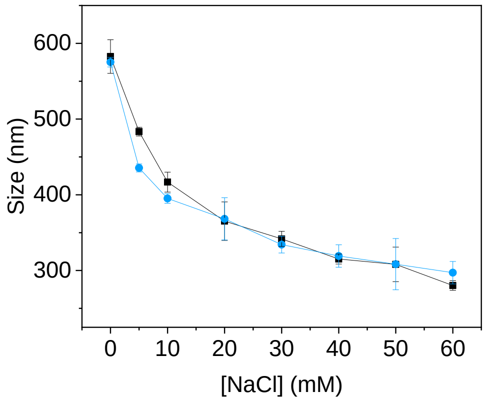

Figure S4. Effect of NaCl, up to 60 mM, on the hydrodynamic diameter of PMVEMA-Es NPs (0.03 M) in absence (black) and in presence (blue) of HTMA-PFP (1.5μM).

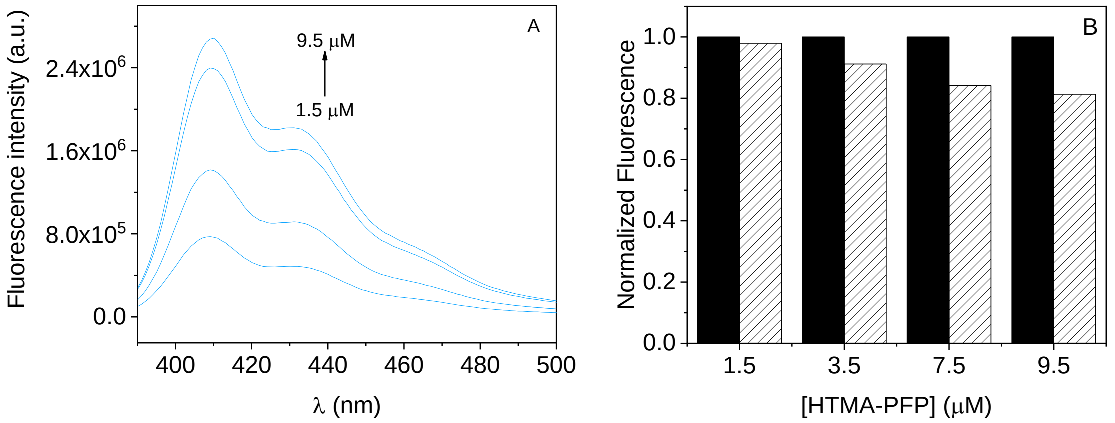

Figure S5. (**A**) Fluorescence emission spectra of HTMA-PFP in PMVEMA-Es NPs (0.03 M) at different concentrations of polyfluorene (1.5, 3.5, 7.5 and 9.5 μM). (**B**) Normalized fluorescence intensity of HTMA-PFP in PMVEMA-Es NPs (0.03 M) at different concentrations (1.5, 3.5, 7.5 and 9.5 μM) recorded the first (black bar) and the fifth day (strip bar) after preparation.

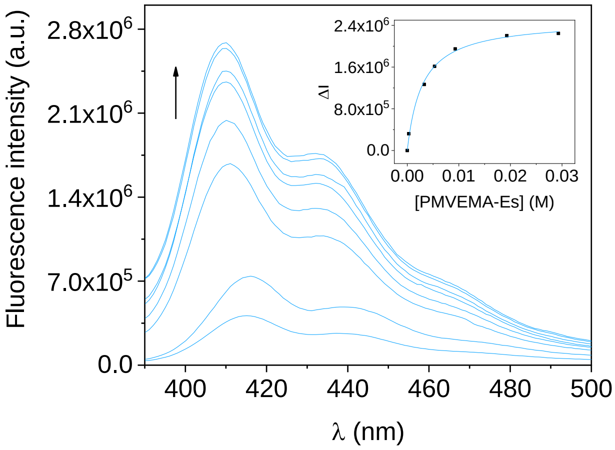

Figure S6. Fluorescence emission spectra of HTMA-PFP (1.5 μM) in MilliQ water with increasing concentrations of NPs (0-0.03 M). Inset: Changes in the fluorescence intensity of the spectrum as a function of PMVEMA-Es concentration.

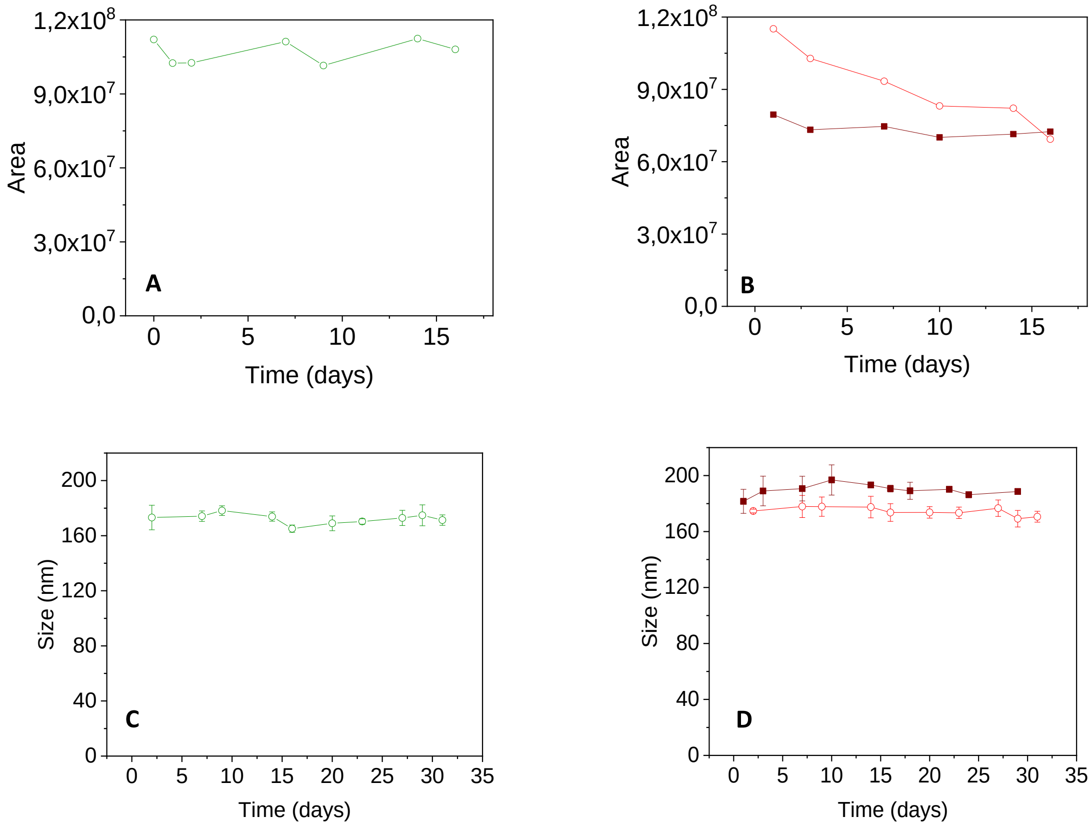

Figure S7. Effect of time storage on the area of the fluorescence emission spectrum (**A-B**) and hydrodynamic diameter (**C-D**) of green (**A**, **C**) and red NPs (**B**, **D**). Open circles and filled squares indicate the addition of polyelectrtolyte after and during the preparation of PMVEMA-Es NPs, respectively.

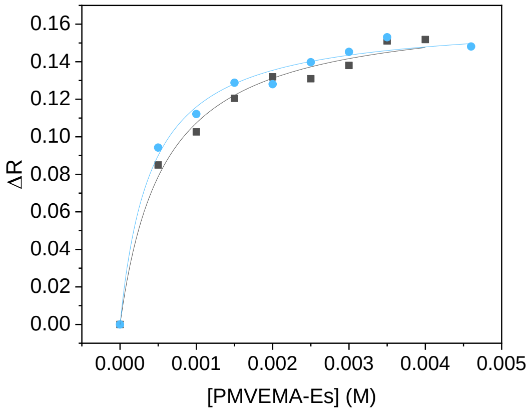

Figure S8. Changes in the ratio of emission peaks, ΔR=(I_{590}/I_{557}) - (I_{590}/I_{557})$_{water}$, of DOX in water at increasing amounts of NPs in presence (blue circles) and in absence (black squares) of HTMA-PFP.

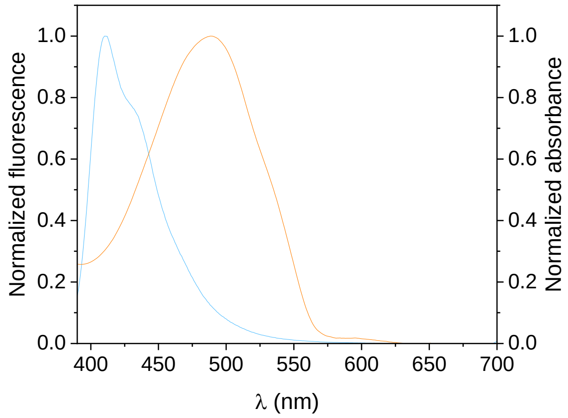

Figure S9. Overlap between the fluorescence emission spectrum of HTMA-PFP (blue) and the absorption spectrum of DOX (orange) in MilliQ water.

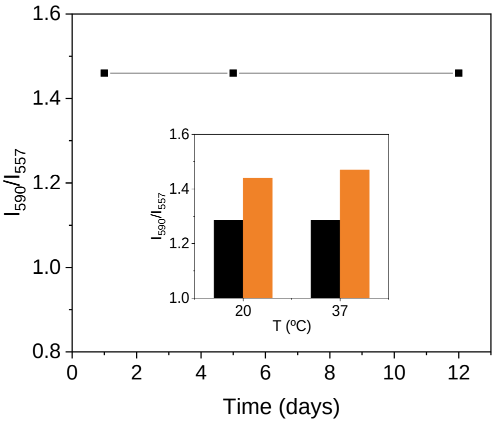

Figure S10. Effect of storage time on the ratio of the intensities of DOX emission peaks, $R=I_{590}/I_{557}$, inserted in the blue-emitting NPs. Inset: Effect of temperature on R, for DOX inserted in fluorescent NPs (orange bars) or free in water (black bars). λ_{exc}=380 nm.

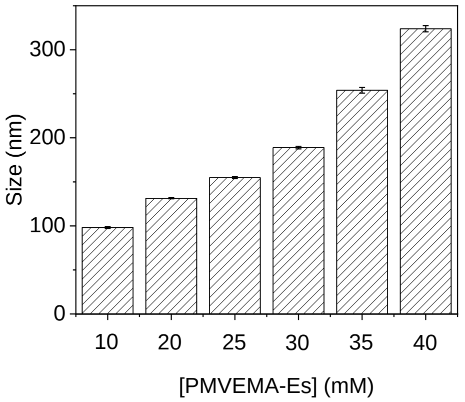

Figure S11. Hydrodynamic diameters, measured by DLS, of NPs prepared in MilliQ water, as a function of PMVEMA-Es concentration.
